# Regulation of gut microbiota-bile acids axis by probiotics in inflammatory bowel disease

**DOI:** 10.3389/fimmu.2022.974305

**Published:** 2022-09-23

**Authors:** Lingfeng Li, Tianyu Liu, Yu Gu, Xinyu Wang, Runxiang Xie, Yue Sun, Bangmao Wang, Hailong Cao

**Affiliations:** Tianjin Key Laboratory of Digestive Diseases, Tianjin Institute of Digestive Diseases, Department of Gastroenterology and Hepatology, Tianjin Medical University General Hospital, Tianjin, China

**Keywords:** inflammatory bowel disease, gut microbiota, bile acids, probiotics, bile salt hydrolase, bile acid-inducible enzymes, intestinal immunity

## Abstract

Inflammatory bowel disease (IBD) is characterized by chronic and relapsing inflammation of gastrointestinal tract, with steadily increased incidence and prevalence worldwide. Although the precise pathogenesis remains unclear, gut microbiota, bile acids (BAs), and aberrant immune response play essential roles in the development of IBD. Lately, gut dysbiosis including certain decreased beneficial bacteria and increased pathogens and aberrant BAs metabolism have been reported in IBD. The bacteria inhabited in human gut have critical functions in BA biotransformation. Patients with active IBD have elevated primary and conjugated BAs and decreased secondary BAs, accompanied by the impaired transformation activities (mainly deconjugation and 7α-dehydroxylation) of gut microbiota. Probiotics have exhibited certain positive effects by different mechanisms in the therapy of IBD. This review discussed the effectiveness of probiotics in certain clinical and animal model studies that might involve in gut microbiota-BAs axis. More importantly, the possible mechanisms of probiotics on regulating gut microbiota-BAs axis in IBD were elucidated, which we focused on the elevated gut bacteria containing bile salt hydrolase or BA-inducible enzymes at genus/species level that might participate in the BA biotransformation. Furthermore, beneficial effects exerted by activation of BA-activated receptors on intestinal immunity were also summarized, which might partially explain the protect effects and mechanisms of probiotics on IBD. Therefore, this review will provide new insights into a better understanding of probiotics in the therapy targeting gut microbiota-BAs axis of IBD.

## 1 Introduction

Inflammatory bowel disease (IBD), which encompasses mainly two clinical phenotypes, was first described in 1895. The two phenotypes are ulcerative colitis (UC) and Crohn’s disease (CD) (1). The epidemiological stages of IBD’s evolution are Emergence, Acceleration in Incidence, Compounding Prevalence, and Prevalence Equilibrium. Western countries are in the third stage, newly industrialized regions are in the second stage, and developing countries are in the first stage as of the end of 2020. In addition, the rising global burden of IBD may be improved by comprehending the changing epidemiological patterns (2). Furthermore, it is of great importance to know the etiology and pathogenesis of IBD. The genetic agents, gut dysbiosis, environmental factors, immunological status, and permeability of gut barrier are closely related to the initiation and development of IBD, even though the precise causes and mechanisms still remain unclear (3). Among them, gut microbiota plays an irreplaceable role as its regulatory and metabolic functions. For instance, they participate in the bile acids (BAs) metabolism to produce unconjugated and secondary BAs for their possession of bile salt hydrolase (BSH) and bile acid–inducible enzymes (BAI) (4). The gut dysbiosis and bile acid dysmetabolism that existed in patients with IBD have been discovered by many studies, the levels of secondary BAs are lower, primary BAs are elevated because of the impairment of microbiota deconjugation and transformation activities, and the microbial BSH activity is decreased (5–7). Therefore, gut microbiota and BAs metabolism play important roles, which have been studied in IBD. In addition, it is widely believed that aberrant immune response against the gut microorganisms in genetically susceptible individuals is the cause of IBD. Immune cell trafficking and some cytokines, such as tumor necrosis factor (TNF), interleukin-10 (IL-10), IL-22, IL-6, IL-17, IL-12, and IL-23 are involved in the immunological pathogenesis of IBD (8). Probiotics, one of the adjuvant therapeutic methods of IBD, have been revealed to be effective in some clinical and animal studies. It can rebalance the aberrant gut microbiota by increasing beneficial microbes that may regulate BAs metabolism and simultaneously inhibiting pathogens (9). Chronic inflammatory, a gut condition in long-term IBD, has an increased risk of developing into intestinal cancer, which is perceived as colitis-associated cancer (CAC) (10). The abnormal BAs metabolism and gut dysbiosis also exist in CAC. The CAC mice model showed gut dysbiosis, decreased fecal BAs, and lessened transformation of primary to secondary BAs (11). Probiotic intervention exhibited inhibition of tumor formation and anti-inflammation effects in CAC mice to some extent (12, 13). Therefore, probiotics may adjust gut bacteria involved in BAs biotransformation in IBD and CAC.

For better understanding the probiotics, in the present review, we focused on the certain effects and probable functional roles of probiotics on regulating gut microbiota-BAs axis in IBD and CAC.

## 2 Microbial influence on BAs in IBD

### 2.1 BAs synthesis and circulation

In gastrointestinal tract, the biotransformation of intestinal BAs is central to the metabolic homeostasis (14). Disorders in BAs metabolism can result in dyslipidemia, cholestatic liver diseases, cardiovascular diseases, fatty liver diseases, diabetes, and so forth (15). Abnormal BAs metabolism detected in IBD may facilitate pro-inflammatory intestinal responses *via* its effects on immune cells and epithelial intestinal cells. The processes of BAs synthesis and circulation have been extensively reviewed elsewhere. BAs synthesize in hepatocytes through the classical and alternative pathways. Primary BAs include cholic acid (CA) and chenodeoxycholic acid (CDCA). Subsequently, CA and CDCA conjugate to either glycine (predominantly in humans) or taurine (mainly in mice) to form conjugated BAs. Then, these BAs are released into intestine after a meal, and approximately 95% of them recirculate through enterohepatic circulation in the distal ileum. The remaining about 5% BAs are transported into colon and further metabolized by the colonic microbiota. The bacteria with BSH activity can deconjugate BAs into their unconjugated forms, including CA and CDCA. Afterward, they are converted to secondary BAs, namely, ursodeoxycholic acid (UDCA), lithocholic acid (LCA), and deoxycholic acid (DCA) through the microbiota possessing enzyme action of 7α-dehydroxylation. In addition, iso-BAs are produced by oxidation and epimerization at C3, C7, and C12 positions of hydroxyl by bacteria with hydroxysteroid dehydrogenases (HSDH) (14, 16).

Crosstalk between BAs and gut microbiota affect metabolic phenotypes, immunological functions, and risk factors for many diseases including diabetes, obesity, non-alcoholic fatty liver disease, IBD, and various cancers (7). In addition to participating in lipid absorption and cholesterol homeostasis, regulating their own biosynthesis, and maintaining a healthy gut microbiota, BAs can also balance carbohydrate metabolism, insulin sensitivity, and innate immunity. The imbalance of BAs production, function, or reabsorption is related to distinct gastrointestinal diseases, such as intestinal inflammation and carcinogenesis, and gastrointestinal motility (5, 14). In IBD patients, although many studies have demonstrated the existence of BAs malabsorption or reduced BAs recycling, both are commonly neglected. Due to decreased microbial abundance in the distal ileum and colon, patients with active IBD had elevated conjugated BAs accumulation and decreased secondary BAs. In addition, the transformation activities that included deconjugation, 7α-dehydroxylation, and desulphation of gut microbiota were impaired (5, 17, 18). Analogously, the same results were observed in many colitis rodent models. In the trinitrobenzene sulfonic acid (TNBS)–induced colitis model, on account of decreased expression of BA transporters, BAs amassing in feces were increased, which resulted in the suppression of BAs recycling (19). Rats with dextran sodium sulfate (DSS)–induced colitis also exhibited accumulation of CA in feces (20). Of note, the microbiome of pediatric patients with IBD was overtly depleted in their BAs production potential (18). Furthermore, in colectomy-treated patients with UC, their pouches had reduced levels of DCA and LCA and lessened genes required to convert primary to secondary BAs (21). With respect to CAC, a study discovered that fecal BAs were lessened, accompanied by decreased transformation of primary to secondary BAs. Moreover, downregulated gut-liver farnesoid X receptor–fibroblast growth factor 15 (FXR-FGF15) axis was also revealed in the CAC mice model (11).

### 2.2 BAs metabolism: Regulatory role of gut microbiota

The synthesis and metabolism of BAs are prominently modified by gut bacteria with diverse enzymes (22). BSH and BAI expressed by certain gut bacteria are the two main enzymes that involve in the deconjugation of primary BAs and subsequent transformation into secondary BAs (4). The major BAs biotransformation in the human gut comprises deconjugation, 7α-dehydroxylation, oxidation and epimerization, desulfatation, and esterification (23). BAs biotransformation is a collaborative effort through the gut microbiota and host. This biotransformation process may play a role in bile detoxification, thus reducing BAs toxicity, and may have beneficial effects on bacteria that carry functional genes. The gut microbiota modulates BAs generation and signalling by the biotransformation of gut BAs to unconjugated and secondary forms that have influence on host health and diseases (24). The major bacterial genera of gut microbiota participated in gut microbiota-BAs axis include *Bifidobacterium*, *Lactobacillus*, *Bacteroides*, *Clostridium*, and *Listeria* in BAs deconjugation; *Clostridium* and *Eubacterium* in 7α-dehydroxylation; *Clostridium*, *Bacteroides*, *Escherichia*, *Eubacterium*, *Egghertella*, *Ruminococcu*, and *Peptostreptococcus* in oxidation and epimerization; *Fusobacterium*, *Clostridium*, *Peptococcus*, and *Pseudomonas* in desulfatation; *Lactobacillus*, *Bacteroides*, and *Eubacterium* in esterification (25). BAs and gut microbiota can influence each other, gut microbiota modulates the size and constitute of the BAs pool, which in turn regulate microbiota composition. Hydrophobic BAs at high concentrations can generate direct antimicrobial activities mainly *via* membrane damage. BAs could also indirectly shape the composition of gut microbiota through BAs receptors. Gram-positive microorganisms are often more sensitive to BAs than Gram-negative microorganisms (7). Nevertheless, BAs could induce the proliferation of some gut microbes. In the context of high fat diet, elevated taurine-conjugated BAs aggrandized the availability of organic sulphur utilized by *Bilophila wadsworthia*, which was associated with increased incidence of colitis in *Il10^−/−^* mice (26).

#### 2.2.1 Deconjugation

In IBD patients, the low level of BSH activity in the microbiota was demonstrated by bioinformatic analysis of metagenomic data (6). BSH, which is oxygen insensitive, is generally located inside the cell, and the optimal pH is commonly between 5 and 6 (27). BSH catalyzes the hydrolysis of conjugated BAs at C24 *N*-acyl amide bond. A number of studies aimed to illustrate the key secondary structure element and amino acids that may be participated in the substrate binding in BSH. These reports exhibited that BSH can identify its substrates through hydrophobic interactions with steroid moiety (28–31). Functional BSH has been identified in all major gut bacteria divisions and archaeal species (32). The main bacteria involved in BAs deconjugation at genera level are *Bifidobacterium*, *Lactobacillus*, *Bacteroides*, *Clostridium*, and *Listeria* (25). Apart from the above five genera, Song and co-workers discovered that *Parabacteroides*, *Bacillus*, *Mycobacterium*, *Staphylococcus*, *Enterococcus*, *Eubacterium*, *Blautia*, *Peptoclostridium*, *Fusobacterium*, *Rhodopseudomonas*, *Yersinia*, and *Vibrio* possessed more than five BSHs (33). It is beneficial for the gut microbiota to modify conjugated BAs, since it may be relevant to the detoxication of conjugated BAs and the acquisition of nitrogen, carbon, and sulfur, which have effect on the growth of bacteria (27, 34). Catalysis of BSH enzymes is the first step in the transition from conjugated BAs to their unconjugated forms, followed by further conversion into secondary BAs. Due to the likely less easily absorbed of secondary BAs, the reabsorption of BAs in the enterohepatic circulation was reduced. Certain BAs may be more easily excreted from individuals, and BA neo-synthesis may be strengthened (35, 36). For instance, as the most hydrophobic BA, LCA is reabsorbed weakly back into enterohepatic cycle, which leads to higher amounts of LCA in feces (37). In the context of chronic inflammation, such as IBD, unconjugated BAs might be gradually exhausted, presumably on account of the depletion of BSH-rich gut bacteria (38). Replenishment of probiotics may improve the decreased BSH in IBD. More importantly, the BSH activity of probiotics may be desirable, because it maximizes its prospects for survival in the harsh environment of intestine, which is likely to enhance the overall beneficial effects related to the strains (27).

#### 2.2.2 7α-dehydroxylation

Only a limited number of intestinal bacteria, including *Clostridium* and *Eubacterium* that encode *bai* genes, can accomplish this process of BAs dehydroxylation. The *bai* genes were significantly reduced in UC than familial adenomatous polyposis pouches (21). Furthermore, BA dehydroxylation can only occur after deconjugation because of the inaccessibility of the hydroxyl group, which is different to oxidation and epimerization, and the 7α-dehydroxylation is the most significant conversion of BAs from quantity and physiology aspects in humans (39). Taking the multi-step *bai* encoded pathway as an illustration, it contains BA import, modification, and export from bacteria. There exist noticeable species-specific discrepancies in the distribution of certain *bai* genes. *Clostridium sordellii* VPI 9048 carries merely *bai*A2, *bai*CD, *bai*E, *bai*H, and 7α-HSDH, whereas *Clostridium hiranonis* carries *bai*J, *bai*H, *bai*BCDEA2FG, and 7α-HSDH (40). Nevertheless, it remains to be elucidated the true distributions of *bai* genes among bacterial species and is still poorly understanding the minimum *bai* gene set that secondary BAs synthesis requires. Moreover, in addition to one advantage that produces reduced nicotinamide adenine dinucleotide phosphate of the *bai* system, other evolutionary advantages are also the subject of speculation and should be further revealed (41).

#### 2.2.3 Oxidation, epimerization, and esterification

HSDHs from intestinal bacteria catalyze the invertible oxidation of hydroxy to oxo groups. Epimerization occurs by stereospecific oxidation and reduction at 3-, 7-, and 12- hydroxyl groups of BAs (42). Epimerization requires the role of two different HSDHs and can be completed *via* a single species with both α- and β-HSDHs or through two species containing α-HSDH and β-HSDH, respectively (43). For instance, 3α/β-HSDH can epimerize DCA to 3-oxo/iso-DCA in *Ruminococcus gnavus* (44). Moreover, allo-BAs are generated by 5-β/α-epimerization (45). Esterified BAs in fecal samples of rodents and humans have been discovered by numerous reports. In addition, esterified BAs may account for more than 25% of total fecal BAs (46).

### 2.3 BAs and intestinal immunity

The disrupted composition, diversity, and/or functions of gut microbiome are closely associated with gut dysbiosis, which has deleterious influence on individuals through gut homeostatic imbalance and inappropriate immune activation (47–49). Although the member and abundance of intestinal microbiota have wide individual differences by taxonomic criteria, there exist relative consistent microbial patterns in the gut of IBD, such as decreased microbial diversity and relative abundance of *Firmicutes*, and increased *Proteobacteria* (50–53). Meanwhile, certain beneficial bacteria are reduced, whereas pathogens including *Escherichia coli* are elevated (54, 55). In the context of IBD, gut dysbiosis and BAs disturbance, especially the reduced secondary BAs, have an impact on intestinal immunity (55). IL-17 and interferon-γ (IFN-γ) are increased on account of the dysregulation of group 3 innate lymphoid cells (ILC3) and ILC1 as well as dysfunction of regulatory ILC (56). The gut microbiota from patients with IBD could decrease retinoic acid receptor related orphan receptor γt (RORγt)^+^ T_reg_ cells that produce transforming growth factor-β (TGF-β) and IL-10, and elevate Th17 (T helper 17) cells with pro-inflammatory cytokines (e.g., IL-17) (57, 58). Bile acid–activated receptors (BARs), a family of nuclear and cell membrane receptors, encompass Takeda G-protein receptor 5 (TGR5), FXR, vitamin D receptor (VDR), pregnane X receptor (PXR), constitutive androstane receptor (CAR), sphingosine 1-phosphate receptor 2, liver-X-receptor α/β, and M2/3 muscarinic receptors (59). Deficiency or inactivation of FXR or TGR5 in macrophages and dendritic cells (DCs) augments the production of pro-inflammatory cytokines. In *FXR^−/−^* mice, infiltration of macrophages was enhanced. In addition, macrophages isolated from TNBS-treated *FXR^−/−^* mice rather than wild-type mice exhibited higher released inflammatory cytokines. The activation of TGR5 in lipopolysaccharide (LPS)–treated macrophages by DCA, LCA, and tauro-LCA was able to inhibit the generation of IL-12 and TNF-α. Simultaneously, it increased the ratio of IL-10/IL-12, indicating the phenotypic transformation of macrophages into anti-inflammatory forms (60). Recruitment of classically activated macrophages and intestinal inflammation was increased in *TGR5^−/−^* mice with colitis. On the contrary, TGR5 activation by agonist could reverse intestinal inflammation through decreasing the trafficking of monocytes from blood to gut (61).

BARs are discovered in intestinal epithelial cells, intestinal muscle and neurons, hepatocytes, biliary cells, liver sinusoidal cells, liver and intestinal endothelial cells, monocytes/macrophages cells, DCs, natural killer (NK) and NKT cells, ILC, Th1, and Th17 cells (62). CDCA (CA in mice) is recognized as the most potent FXR ligand in humans, followed by DCA, LCA, and CA, whereas secondary BAs, particularly LCA and DCA, are preferential ligands for the TGR5, whose other ligands are CDCA, UDCA, and CA (59). VDR is activated by LCA and its metabolites (isoallo-LCA and 3-oxo-LCA). Furthermore, isoallo-LCA and 3-oxo-LCA can act on RORγt as inverse agonists in DCs and T cells (63). We hereafter briefly summarized the main functions of BARs activation in different immune cells. In monocyte/macrophages cells, the activation of FXR reduces the generation of TNF-α and IL-1β under inflammatory conditions. TGR5 activation shifts intestinal macrophages that are treated with LPS from M1 to M2; decreases the expression of TNF-α, IL-1β, IFN-γ, IL-6, and IL-12; and elevates the level of IL-10. In addition, VDR activation inhibits the release of IL-1, IL-6, and TNF-α but enhances IL-10 production (61, 64, 65). As far as DCs, FXR activation inhibits the differentiation, activation, and maturation of intestinal DCs and downregulates TNF-α expression. In addition, as stated previously, the activation of TGR5 in DCs reduces the production of IL-12 and TNF-α. Suppression of DCs differentiation and maturation by activating VDR has been demonstrated (66–68). In ILC3, RORγt is required for its development and function (69, 70). In NKT cells, the activation of FXR could repress the production of IFN-γ and osteopontin. TGR5 activation redirects the NKT cells polarization toward NKT10, subsequently promoting the secretion of IL-10 whereas decreasing the TNF-α and IFN-γ (71, 72). Nonetheless, the effect of FXR/TGR5 activation in NKT cells has been investigated only in the liver. These results may indicate a similar effect of them on intestinal NKT cells, which needs to be further explored. As for T cells, by directly binding to the RORγt in Th17 cell, 3-oxo-LCA and isoLCA inhibit Th17 differentiation (73). Recently, LCA 3-sulfate, a synthesized sulfated product of LCA, suppresses Th17 cell differentiation through binding to RORγt (74). IsoalloLCA can increase the differentiation of T_reg_ cells (75). IsoDCA acts on FXR in DCs and then results in the promotion of RORγt^+^FOXP3^+^ T_reg_ cells (76). Beyond this, TGR5 agonism promotes T-cell differentiation toward the T_reg_ phenotype (61). VDR activation facilitates a shift from Th1 to Th2 phenotype, inhibits proliferation of T cell and Th17 differentiation, and promotes the induction of T_reg_ cells (77–81). Therefore, the anti-inflammatory and immunomodulatory effects of BAs-BARs-immune cell axis play essential roles in health and disease conditions.

## 3 Effectiveness of probiotics and their possible roles in gut microbiota-BAs axis

In accordance with the World Health Organization, probiotic bacteria are defined as live microorganisms that confer a health benefit to the host when administered in adequate amounts. Probiotic strains must have four criteria for selection and use in foods or dietary supplements. In brief, first, they must be sufficiently characterized. Second, safe use is an essential requirement. In addition, at least one human clinical trial can support their positive effectiveness by using probiotics. Last but not the least, it must be alive at an efficacious dose in the product during the quality guarantee period (82). The majority of probiotics used in research or commercial development are from limited bacteria, which mainly contain *Bifidobacterium* spp. and *Lactobacillus* spp. Currently, other probiotics, such as *Saccharomyces*, *E. coli*, *Bacillus* spp., *Weissella* spp., and *Enterococci* are available in the marketplace (83).

### 3.1 Effectiveness of probiotics on IBD/CAC

#### 3.1.1 Probiotic effectiveness in clinical trials among IBD patients

As of now, some clinical trials have been presented the positive effects of probiotics on patients with IBD. Next, we listed some clinical trials and the altered fecal bacteria that might participate in the metabolism of BAs. In UC patients, administration of probiotic yogurt, which included *Lactobacillus acidophilus* La-5 and *Bifidobacterium* BB-12, led to increased numbers of *Bifidobacterium*, *Lactobacillus* and *Bacteroides* in feces (84). These three bacteria (genus level) might regulate the gut microbiota-BAs axis due to their BSH activities or other functions. BIFICO (*Bifidobacterium longum*, *Lactobacillus acidophilus*, and *Enterococcus faecalis*) prevented flare-ups of chronic UC by decreasing IL-1β and TNF-α levels, increasing IL-10 expression, and blocking Nuclear Factor kappa-light-chain-enhancer of activated B cells (NF-κB) activation. Moreover, it elevated fecal *Lactobacillus* and *Bifidobacterium* (85). Similar fecal bacteria variations were also found in another UC trials with BIFICO (86). Furthermore, mesalazine combined with BIFICO treatment reduced the adverse reactions (87). In a 2-year clinical study, mesalazine plus a probiotic mix improved the clinical response better than controls (88). Tsuda et al. showed that by making use of BIO-THREE (*Streptococcus faecalis* T-110, *Clostridium butyricum* TO-A, and *Bacillus mesentericus* TO-A) ameliorated endoscopic findings, clinical symptoms of UC patients, and increased fecal *Bifidobacterium* (89). In addition to these, many other studies also exhibited the effectiveness of the same or diverse probiotics on inducing remission or preventing recurrence of IBD patients (90–97). Moreover, application of *Escherichia coli* Nissle 1917 (EcN) by rectum was a well replacement treatment for moderate distal UC (98). Among CD patients, *Saccharomyces boulardii* was useful for maintenance treatment according to a clinical trial in 2000 (99).

Nevertheless, there exist some discrepant results of using probiotics in IBD. For example, *Bifidobacterium breve* fermented milk had no effect on relieving relapse in UC (100). In a small randomized double-blind placebo-controlled trial of UC, *Lactobacillus acidophilus* La-5 and *Bifidobacterium animalis* subsp. *lactis* BB-12 showed no significant clinical benefit (101). In another clinical trial, EcN did not improve the active UC followed by 7 weeks administration after ciprofloxacin or placebo intervention (102). With regard to CD, Bourreille et al. found that after steroid or salicylate treatment, *Saccharomyces boulardii* had no beneficial effect on CD patients in remission (103). Van Gossum et al. did not find that *Lactobacillus johnsonii* LA1 could prevent early endoscopic recurrence of CD patients after ileo-caecal resection (104). The reasons for why the diverse outcomes appear are intricate. It may attribute to strain-specific, dose, utility time, number of patients, individual differences, disease severity, microbiota composition, genetic aspects, inflammatory status, and so forth.

Hence, from the valid clinical trials of IBD, we can discover that the main elevated fecal bacteria (genus level) that may involve in modulating gut microbiota-BAs axis are *Lactobacillus* and *Bifidobacterium*. Only one clinical trial found the relative increased *Bacteroides*. Moreover, the outcomes and other effects including immune response of these clinical trials were also presented in **Table 1**.

**Table 1:** Probiotics modulate immune response and the gut bacteria that may be involved in gut microbiota-bile acids axis in patients with inflammatory bowel disease.

Author[year]	Probiotic	Patients	Dose and duration	Detection method	Outcomes	Elevated relevant microbes that may be involved in gut microbiota-bile acids axis(genus/species)	Other effects
Cui *et al.* [2004]	BIFICO (85)	30 with UC	1.26 g/day, 8 weeks	Culture	↓ recurrence rate	*Lactobacillus*, *Bifidobacterium*	↓IL-1β, TNF-α; ↑IL-10; ↓ NF-κB
Tsuda *et al.* [2007]	BIO-THREE (89)	20 with UC	9 BIO-THREE tablets, 4 weeks	T-RFLP	Improved UCDAI score	*Bifidobacteria*	–
D’Inca *et al.* [2011]	*Lactobacillus casei* DG (105)	26 with UC	8*10^8^ CFU (twice daily), 8 weeks	Culture	Improved histological disease severity scores, which were directly correlated with TLR4 mRNA mucosal levels	*Lactobacillus* spp.	↓IL-1β, TLR4; ↑IL-10
Shadnoush *et al.* [2015]	*Lactobacillus acidophilus* La-5 and *Bifidobacterium* BB-12 (84)	198 with UC, 22 with CD	250g/day (10^6^ CFU/g), 8 weeks	qPCR	–	*Lactobacillus*, *Bifidobacterium*, and *Bacteroides*	–
Fan *et al.* [2019]	BIFICO (86)	40 with IBD	2 probiotics tablets (three times a day), 40 days	Culture	↓CDAI, UCAI, and recurrence rate ↓lactoferrin, α- antitrypsin, β2-microglobulin, and hs-CRP	*Bifidobacterium*, *Lactobacillus*	↓IL-6; ↑IL-4

IBD, inflammatory bowel disease; CD, Crohn’s disease; UC, ulcerative colitis; CFU, colony forming units; TNF, tumor necrosis factor; IL, interleukin; NF-κB, Nuclear Factor kappa-light-chain-enhancer of activated B cells; TLR, toll-like receptor; CDAI, Crohn’s disease activity index; UCAI, UC activity index; hs-CRP, high-sensitivity C-reactive protein; UCDAI, ulcerative colitis disease activity index; T-RFLP, terminal restriction fragment length polymorphism; qPCR, quantitative real-time polymerase chain reaction.

* means the mathematical symbol ×; ↓ means reduced; ↑ means increased.

#### 3.1.2 Probiotic effectiveness in animal models with colitis

White and co-workers demonstrated that VisbiomeTM (*Lactobacillus plantarum*, *Streptococcus thermophilus*, *Bifidobacterium breve*, *Lactobacillus paracasei*, *Lactobacillus delbrueckii* subsp. *bulgaricus*, *Lactobacillus acidophilus*, *Bifidobacterium longum*, and *Bifidobacterium infantis*) plus prednisone therapy could accelerate clinical remission, upregulate the expression of E-cadherin, occludin, and zonulin but did not improve the histopathologic inflammation than prednisone alone group in canine with idiopathic IBD. In addition, the combination therapy increased fecal *Bifidobacterium* (106). In the TNBS-induced colitis model, *Lactobacillus plantarum* LC27 and *Bifidobacterium longum* LC67 inhibited *Proteobacteria* to *Bacteroidetes* ratio and NF-κB activation, elevated fecal *Lactobacilli*, *Bifidobacteria* and tight junction protein expression, simultaneously restored Th17/T_reg_ balance (107). Another study using *Lactobacillus acidophilus* and *Clostridium butyricum* acquired similar results of elevated fecal *Lactobacillus* and *Bifidobacterium*. Furthermore, it alleviated reduced colon length and body weight, suppressed disease activity indices (DAI), and exerted an anti-inflammatory effect (108). VSL#3 (*Lactobacillus paracasei*, *L. plantarum*, *L. acidophilus*, *L. delbrueckii* subspecies *bulgaricus*, *Bifidobacterium longum*, *B. breve*, *B. infantis*, and *Streptococcus thermophilus*) lowered macroscopic and microscopic damage, reduced serum cytokine levels, but not dampening M1 macrophages. Of note, fecal *Parabacteroides*, *Clostridium* were increased in mice being given VSL#3 than in the TNBS colitis mice (109).

Within the DSS-induced colitis model, *Lactobacillus rhamnosus* GG (*LGG*) ameliorated decreased body weight and clinical features and simultaneously enhanced fecal *Lactobacillus* and *Bacteroides* (110). Three studies revealed the positive effect of *Lactobacillus plantarum* on DSS-induced colitis, *Lactobacillus plantarum* GIM17 and *Lactobacillus plantarum*-12 elevated the fecal *Lactobacillus*, whereas the *Lactobacillus plantarum* L15 could also increase other bacteria, such as *Bifidobacterium* and *Bacteroides* (111–113). *LGG* and *Lactobacillus plantarum* Q7–derived extracellular vesicles could also enhance the fecal *Bifidobacterium_animalis* or *Lactobacillus* and *Bifidobacterium*, respectively (114, 115). Jang et al. and Zhang et al. discovered that *Lactobacillus fermentum* KBL375 or *Lactobacillus casei* Zhang was capable of augmenting species levels of *Lactobacillus* in feces (116, 117). *Lactobacillus* M2S01 recovered decreased body weight and incremental DAI, promoted anti-inflammatory cytokines expression, and elevated fecal *Bifidobacterium* (118). Higher *Lactobacilli*, *Bifidobacteria*, or *Bacteroides* in feces were detected after the administration of *Lactobacillus salivarius* CPN60 or *Lactobacillus brevis*, respectively (111, 119). As for *Bifidobacterium longum* CCFM681, Chen et al. demonstrated the protective roles of it in mice with DSS-induced colitis. It significantly increased mucin 2, goblet cells, zonula occludens-1 (ZO-1), claudin-3 and α-catenin1, suppressed Toll-like receptor 4/NF-κB pathway and related pro-inflammatory cytokines. Moreover, it promoted the growth of *Lactobacillus* and *Bifidobacterium* in feces (120). The authors also discovered that *Bifidobacterium pseudocatenulatum* MY40C and CCFM680 were able to elevate fecal *Lactobacillus* as well (121). Oral delivery of *Bacillus cereus* or *Bacillus subtilis* alleviated DSS-induced colitis through anti-inflammation, protecting intestinal integrity, improving intestinal barrier function, and reshaping microbial composition, which comprised increased fecal *Lactobacillus*, *Eubacterium*, *Bacillus*, and *Bacteroides* (122–125). Rodriguez-Nogales et al. found that *Saccharomyces boulardii* CNCMI-745 improved the colitis-associated gut dysbiosis, including more *Lactobacillus*, *Bifidobacterium* in feces (126). *Saccharomyces cerevisiae* BR14 merely increased the fecal *Lactobacillus* (127), whereas an engineered strain reconstructed from *Saccharomyces cerevisiae* BY4741 could also enhance fecal *Bacteroides* apart from *Lactobacillus* in DSS-induced colitis (128). The aforementioned research revealed the potential functions and part mechanisms of single probiotic on animals with DSS-induced colitis. Noteworthy is that they are all involved in the alterations of fecal microbiota, which may modulate gut microbiota-BAs axis. Furthermore, numerous other researchers investigated the effectiveness of multi-strain or combined probiotics on the DSS model. *Lactobacillus rhamnosus* R0011 and *Lactobacillus helveticus* R0052 administration resulted in diminished colon disease and an increase in fecal *Bacillus* and *Lactobacillus* (129). Rodriguez-Nogales et al. uncovered the functional roles of *Lactobacillus fermentum* CECT5716 and *Lactobacillus salivarius* CECT5713 on DSS mouse colitis. Both probiotics contributed to anti-inflammatory effects, regulating immune response, improving microRNA (miR)-155 and miR-223 expression, and increasing fecal *Parabacteroides*, *Bacillus* (130). *Lactobacillus sakei* 07 and *Bifidobacterium bifidum* B10 combination decreased colonic IL-6, TNF-α, and intestinal permeability and increased intestinal flora biodiversity and the level of fecal *Bifidobacterium* in the colitis model (131). Quadruple probiotics were also showed benefit influences on colitis mice induced by DSS. *Bifidobacterium infantis* GMCC0460.1, *Lactobacillus acidophilus* GMCC0460.2, *Enterococcus faecalis* GMCC0460.3 and *Bacillus cereus* GMCC0460.4 repaired multi-barriers in the inflamed gut and enhanced *Lactobacillus*, *Bifidobacterium*, and *Bacteroides* in feces (132). Wang et al. uncovered that supplement of *Lactobacillus reuteri* RAM0101, *Bacillus coagulans* RAM1202, *Bifidobacterium longum* RAM0216, and *Clostridium butyricum* CICC6197 increased intestinal barrier function, IL-10 expression and fecal *Bifidobacterium*, *Blautia*, *Lactobacillus*, and *Bacillus coagulans* (133). Analogously, there exist some other new or potential probiotics, such as *Faecalibacterium prausnitzii*, *Pediococcus pentosaceus*, *Ligilactobacillus salivarius*, *Lactiplantibacillus plantarum*, and *Akkermansia muciniphila* have been certified effectively in DiNitroBenzene Sulfonic/DSS-induced colitis model. *Lactobacillus*, *Bacteroides*, *Bifidobacterium*, *Parabacteroides*, and *Eubacterium_fissicatena_group* were increased in feces after using these probiotics (134–138).

In short, the probiotics mentioned above bring forth favorable therapeutic effects on animal models with chemical-induced colitis. Probiotics may regulate gut microbiota-BAs axis by elevating the fecal concentration of *Lactobacillus*, *Bifidobacterium*, *Bacteroides*, *Parabacteroides*, *Clostridium*, *Blautia*, *Bacillus coagulans*, *Eubacterium* or *Eubacterium_fissicatena_group* at genus or species levels. Moreover, other effects involved in immune response were summarized in **Table 2** as well.

**Table 2:** Probiotics modulate immune response and the gut bacteria that may be involved in gut microbiota-bile acids axis in animal models with colitis-associated cancer.

Probiotic	Animal models	Detection method	Elevated relevant microbes that may be involved in gut microbiota-bile acids axis(genus/species)	Other effects
*Lactobacillus rhamnosus* M9 (12)	AOM/DSS-induced CAC	Metagenomic Sequencing	*Blautia*, *Bifidobacterium*	↓PCNA^+^ cells, p-STAT3, p-Akt; ↑Ki67; ↓M1 (CD68^+^) and M2 (CD163^+^) in the serosa
*Lactobacillus fermentum* V3 (139)	AOM/DSS-induced CAC	16S rRNA	*Lactobacillus*	↓IL-1α/β, IL-6, M1 (CD68^+^)
*Lactobacillus casei* BL23 (140)	AOM/DSS-induced CAC	16S rRNA	*Lactobacillus*	↓IL-22, Ki67; ↑caspase-7, caspase-9, Bik
*Lactobacillus gasseri* 505 (141)	AOM/DSS-induced CAC	16S rRNA	*Lactobacillus*	↓IL-1β, TNF-α, IFN-γ, and↑IL-10 (protein level); ↓IL-6, and↑IL-4, IL-10 (mRNA level); ↓iNOS, COX-2, Bcl-xl; ↑MUC2, ZO-1, occludin, p53, p21, Bax
*Lactobacillus coryniformis* MXJ32 (13)	AOM/DSS-induced CAC	16S rRNA	*Lactobacillus*, *Bifidobacterium*	↓IL-1β, IL-6, IL-17a, IL-γ, TNF-α, Cxcl1/2/3/5, Ccl7; ↑ZO-1, occludin, claudin-1, goblet cells, SCFAs
*Lactobacillus helveticus* NS8 (142)	AOM/DSS-induced CAC	16S rRNA	*Parabacteroides;* *Parabacteroides* sp., *Bacteroides acidifaciens*, *Bifidobacterium pseudolongum*	↑IL-10, caspase-3; ↓NF-κB; ↓IL-1β, IL-8, TNF-α, angiogenin, β-catenin, COX-2, Ki67, IL-17-producing T cells
*Bifidobacterium bifidum* CGMCC 15068 (143)	AOM/DSS-induced CAC	16S rRNA	*Lactobacillus*	–
*Clostridium butyricum* (144)	AOM/DSS-induced CAC	16S rRNA	*Lactobacillus*, *Bifidobacterium*	↓IL-6, TNF-α, COX-2, Bcl-2, Ki67; ↑Bax; ↓NF-κB and proliferation
*Lactobacillus acidophilus*, *Lactobacillus rhamnosus* and *Bifidobacterium bifidum* (145)	AOM/DSS-induced CAC	16S rRNA	*Lactobacillus*, *Bifidobacterium*, *Clostridium* XI, *Clostridium* XVIII	↓RANTES, Eotaxin (serum); ↓p-IKK, TNF-α; ↑IL-10
VSL#3 (146)	AOM/DSS-induced CAC	16S rRNA	*Lactobacillus*, *Bacillus*	↓IL-6, TNF-α

CAC, colitis-associated cancer; AOM, azoxymethane; DSS, dextran sodium sulfate; PCNA, proliferating cell nuclear antigen; p-STAT3, phosphorylated signal transducer and activator of transcription 3; p-Akt, phosphorylated serine/threonine kinase protein kinase B; M, macrophage; IL, interleukin; Bik, Bcl-2 interacting killer; TNF, tumor necrosis factor; IFN-γ, interferon-γ; iNOS, inducible nitrogen oxide synthase; COX-2, cyclooxygenase 2; Bcl-xL, B-cell lymphomaextra-large; MUC2, mucin2; ZO-1, zona occludens-1; Bax, Bcl2-associated X protein; Cxcl, chemotactic factors chemokine ligand; Ccl7, C-C motif ligand 7; SCFAs, short−chain fatty acids; NF-κB, Nuclear Factor kappa-light-chain-enhancer of activated B cells; Bcl-2, B-cell lymphoma 2; IKK, IκB kinase; 16S rRNA, 16S ribosomal ribonucleic acid.

↓ means reduced; ↑ means increased.

#### 3.1.3 Probiotic effectiveness in AOM/DSS induced CAC

Xu and co-workers indicated that *Lactobacillus rhamnosus* M9 suppressed the increased number and growth of colon tumors and expression of phosphorylated-signal transducer and activator of transcription 3 and phosphorylated-protein kinase B, ameliorated inflammatory damage and gut fibrosis in azoxymethane (AOM)/DSS-induced CAC. Furthermore, it changed fecal microbiota, which included elevated *Blautia* and *Bifidobacterium* at genus level (12). *Lactobacillus fermentum* V3 markedly inhibited colonic tumor formation and pro-inflammatory cytokines, accompanied by increased fecal *Lactobacillus* (139). The same fecal bacteria alteration that might participate in gut microbiota-BAs axis was also observed in AOM/DSS-induced CAC mice by using *Lactobacillus casei* BL23 or *Lactobacillus gasseri* 505. Other than this function, *Lactobacillus casei* BL23 reduced proliferation and histological scores, downregulated IL-22 cytokine, and upregulated caspase-7 and caspase-9 (140, 141). Wang et al. revealed that *Lactobacillus coryniformis* MXJ32 could significantly suppress the total number and average diameter of tumors, reinforce the expression of ZO-1, Claudin-1, Occludin, and recover the lesion of goblet cells. In addition, it lowered the expression of IL-1β, TNF-α, IL-6, IL-17a, C-C motif ligand 7, chemotactic factors chemokine ligand 5 (Cxcl5), Cxcl3, Cxcl2 and Cxcl1, increased the abundance of *Lactobacillus*, *Bifidobacterium* in the stool (13). Treatment with *Lactobacillus helveticus* NS8 overtly reduced the degree of hyperplasia and tumor number, suppressed enterocytes proliferation at the early stage of CAC, while it increased apoptosis level. Furthermore, NS8 significantly inhibited NF-κB activation, IL-17–producing T cells, and upregulated IL-10. Interestingly, at the genus level, fecal *Parabacteroides* was augmented after 80 days injection of AOM, while at the species level, *Lactobacillus* sp., *Bacteroides acidifaciens* or *Parabacteroides* sp., *Bifidobacterium pseudolongum* were increased at 14 or 80 days behind AOM injection (142). Wang and co-authors discovered the conducive role of *Bifidobacterium bifidum* CGMCC 15068 on the CAC mouse model. This probiotic was capable of attenuating tumorigenesis and shifting gut microbiota composition, which comprised a higher quantity of *Lactobacillus* in feces. In addition, the authors found the existence of differentially abundant metabolites between AOM/probiotic and AOM groups, which indicated that *Bifidobacterium bifidum* CGMCC 15068 was involved in multiple metabolic pathways, such as citrate cycle, galactose metabolism, butyrate metabolism, and so on (143). *Clostridium butyricum*, a relative new probiotic, has been identified to inhibit the incidence and size of tumors in CAC mice, decrease IL-6, TNF-α, cyclooxygenase-2, phosphorylation of NF-κB and B-cell lymphoma-2. Moreover, it aggrandized the expression of Bcl-2-associated X and fecal *Lactobacillus*, *Bifidobacterium* (144). Triple or multiple probiotics intervention still exhibited a satisfactory effect on the chemical-induced CAC model. *Lactobacillus acidophilus*, *Lactobacillus rhamnosus*, and *Bifidobacterium bifidum* mixture restrained the size and number of tumors, reduced colon inflammatory index, serum chemokines RANTES, eotaxin, phospho-IκB kinase, and TNF-α, whereas it enhanced IL-10 expression and fecal *Lactobacillus*, *Bifidobacterium*, *Clostridium* XI, and *Clostridium* XVIII (145). By making use of VSL#3 in AOM/DSS-induced CAC mice, Wang et al. revealed that this probiotic alone ameliorated oncogenesis and tumor load, and reduced the level of IL-6 and TNF-α in colon tissue. Furthermore, it increased fecal *Bacillus* and *Lactobacillus* as well, but the latter had no statistical significance (146).

As mentioned above, probiotics can improve the AOM/DSS-induced CAC model in a variety of ways, one of which is altering gut microbiota composition. In conclusion, the increased fecal bacteria that may involve in gut microbiota-BAs axis comprise *Blautia*, *Bifidobacterium*, *Lactobacillus*, *Parabacteroides*, *Bacillus*, *Parabacteroides* sp., *Bacteroides acidifaciens*, *Bifidobacterium pseudolongum*, *Clostridium* XI and *Clostridium* XVIII at genus/species level. In addition, other effects containing immune response after treatment with probiotics in CAC model were also showed in **Table 3**.

**Table 3:** Probiotics modulate immune response and the gut bacteria that may be involved in gut microbiota-bile acids axis in animal models with inflammatory bowel disease.

Probiotic	Animal models	Detection method	Elevated relevant microbes that may be involved in gut microbiota-bile acids axis(genus/species)	Other effects
VisbiomeTM (106)	34 dogs with idiopathic IBD	qPCR	*Bifidobacterium*	↑E-cadherin, occluding, zonulin
*Lactobacillus plantarum* LC27 and *Bifidobacterium longum* LC67 (107)	TNBS-induced colitis	qPCR	*Lactobacilli*, *Bifidobacteria*	↓iNOS, COX-2, TNF-α, IL-1β, IL-17, RORγt; ↑IL-10, Foxp3, TJP; Restore Th17/T_reg_ balance
*Lactobacillus acidophilus* CGMCC 7282, *Clostridium butyricum* CGMCC 7281 (108)	TNBS-induced colitis	Culture	*Lactobacillus*, *Bifidobacterium*	↓IL-8, TNF-α; ↑ZO-1; ↓ NF-κB
VSL#3 (109)	TNBS-induced colitis	Fecal metagenomics	*Parabacteroides*, *Clostridium*	↑IL-12
*Lactobacillus rhamnosus* GG (110)	DSS-induced colitis	16S rRNA	*Lactobacillus*, *Bacteroides*	↓IL-6, IL-10 in serum
*Lactobacillus brevis* (111)	DSS-induced colitis	16S rRNA	*Bacteroides*	–
*Lactobacillus plantarum* GIM17 (147)	DSS-induced colitis	16S rRNA	*Lactobacillus*	–
*Lactobacillus plantarum* L15 (112)	DSS-induced colitis	16S rRNA	*Lactobacillus*, *Bifidobacterium*, *Bacteroides*	↓IL-1β, IL-12, TNF-α; ↑IL-10; ↓TLR4-MyD88-NF-κB
*Lactobacillus plantarum*-12 (113)	DSS-induced colitis	16S rRNA	*Lactobacillus*	↓IL-8, TNF-α; ↑IL-10, MUC2
*Lactobacillus reuteri* I5007 (148)	DSS-induced colitis	16S rRNA	*Bifidobacterium, Clostridium*_XIII	↓IL-1β, IL-6, TNF-α, IL-17A
*Lactobacillus casei* Zhang (116)	DSS-induced colitis	16S rRNA	*Lactobacillus reuteri*	↓IL-6(serum), MPO(colon), p-STAT3 signaling
*Lactobacillus fermentum* KBL375 (117)	DSS-induced colitis	16S rRNA	*Lactobacillus* spp.	↓IL-2, IL-4, IL-13, IL-17A; ↑IL-10, T_reg_
*Lactobacillus* M2S01 (118)	DSS-induced colitis	16S rRNA	*Bifidobacterium*	↑IL-10, IL-22; ↓NF-κB
*Lactobacillus salivarius* CPN60 (119)	DSS-induced colitis	Culture	*Lactobacilli*, *Bifidobacteria*	↑lactate, acetate, propionate, butyrate
*Bifidobacterium longum* CCFM681 (120)	DSS-induced colitis	16S rRNA	*Lactobacillus*, *Bifidobacterium*	↓IL-6, MPO; ↓TLR4-NF-κB; ↑IL-10, MUC2, goblet cells, ZO-1, α-catenin1, claudin-3
*Bifidobacterium pseudocatenulatum* MY40C and CCFM680 (121)	DSS-induced colitis	16S rRNA	*Lactobacillus;* *Blautia* was the key microbe in MY40C groups	↓IL-6, TNF-α, MPO, COX-2, Caspase-3; ↓TLR4-NF-κB; ↑IL-10, MUC2, goblet cells, ZO-1, β-catenin, claudin-3
*Bacillus cereus* JNFE0126 (122)	DSS-induced colitis	16S rRNA	*Bacillus*, *Lactobacillus*	↓TNF-α, MPO; ↑IL-10, Lgr5^+^ stem cells, CDX2, MUC2, ZO-1, villine
*Bacillus cereus* HMPM18123 (125)	DSS-induced colitis	16S rRNA	*Lactobacillus*, *Eubacterium*	↓IL-1β, IL-6, TNF-α; ↓TLR4-NF-κB-NLRP3; M1→M2; ↑IL-10, MUC2, goblet cells, ZO-1, occludin, claudin-1
*Bacillus subtilis* (123)	DSS-induced colitis	16S rRNA	*Bacteroides*	↓Ki67; ↑ZO-1, occludin
*Bacillus subtilis* R179 (124)	DSS-induced colitis	16S rRNA	*Bifidobacterium* sp., *Lactobacillus* sp.	↓IL-12, IL-17, IL-23; ↑IL-10 (serum); ↑ZO-1, claudin
**Probiotic**	**Animal models**	**Detection method**	**Elevated relevant microbes that may be involved in gut microbiota-bile acids axis (genus/species)**	**Other effects**
*Saccharomyces boulardii* CNCMI-745 (126)	DSS-induced colitis	16S rRNA	*Lactobacillus*, *Bifidobacterium*	↓IL-1β; ↑TGF-β, MUC2/3, ZO-1, occludin
*Saccharomyces cerevisiae* BR14 (127)	DSS-induced colitis	16S rRNA	*Lactobacillus*	↓IL-6, TNF-α; ↑IL-10
*Lactobacillus fermentum* CECT5716, *Lactobacillus salivarius* CECT5713 (130)	DSS-induced colitis	16S rRNA	*Parabacteroides*, *Bacillus*	↓IL-1β; ↑TGF-β, MUC2/3, ZO-1, occludin, lactate, acetate, propionate
*Lactobacillus sakei* 07 and *Bifidobacterium bifidum* B10 (131)	DSS-induced colitis	16S rRNA	*Bifidobacterium*	↓IL-6, TNF-α, LPS; ↑IL-10, TGF-β
*Bifidobacterium infantis* GMCC0460.1, *Lactobacillus acidophilus* GMCC0460.2, *Enterococcus faecalis* GMCC0460.3 and *Bacillus cereus* GMCC0460.4 (132)	DSS-induced colitis	16S rRNA	*Lactobacillus*, *Bifidobacterium*, and *Bacteroides*	↓IL-1β, TNF-α, intestinal permeability; ↑IL-10, goblet cells, mucus thickness, ZO-1, occludin
*Lactobacillus reuteri* RAM0101, *Bacillus coagulans* RAM1202, *Bifidobacterium longum* RAM0216, and *Clostridium butyricum* CICC6197 (133)	DSS-induced colitis	16S rRNA	*Bifidobacterium*, *Blautia*, *Lactobacillus*, *Bacillus coagulans*	↓IL-1β, IL-6, TNF-α; ↑IL-10, occludin, claudin-1
*Faecalibacterium prausnitzii* A2-165 (135)	Severe/Moderate DNBS-induced colitis	qPCR	*Lactobacillus*, *Bacteroides/* *Lactobacillus*	↓IL-6, IL-12, TNF-α, IFN-γ; ↑T_reg_
*Ligilactobacillus salivarius* Li01 (136)	DSS-induced colitis	16S rRNA	*Bifidobacterium*, *Bacteroides*	↑IL-10 (plasma)
*Pediococcus pentosaceus* CECT 8330 (137)	DSS-induced colitis	16S rRNA	*Lactobacillus*, *Bifidobacterium*	↓IL-1β, IL-6, TNF-α, and↑IL-10 (serum); ↑ZO-1, occludin, T_reg_, acetate, propionate, butyrate
*Lactiplantibacillus plantarum* DMDL 9010 (134)	DSS-induced colitis	16S rRNA	*Bacteroides*, *Lactobacillus*, *Parabacteroides*, *Eubacterium_fissicatena_group*	↓IL-1β, TNF-α, and↑TGF-β (serum); ↑propionate, butyrate
*Akkermansia muciniphila* Muc^T^ (138)	DSS-induced colitis	16S rRNA	*Lactobacillus*	↓IL-12A, TNF-α, IFN-γ, and↑IL-10 (colon); ↓IL-1α, IL-6, IL-12A, TNF-α, MIP-1α, G-CSF, KC, and↑IL-10 (serum); ↑ZO-1, occludin, SCFAs
Extracellular vesicles derived from *Lactobacillus rhamnosus* GG (114)	DSS-induced colitis	16S rRNA	*Bifidobacterium_animalis*	↓IL-1β, IL-2, IL-6, TNF-α; ↓TLR4-NF-κB-NLRP3
Extracellular vesicles derived from *Lactobacillus plantarum* Q7 (115)	DSS-induced colitis	16S rRNA	*Lactobacillus*, *Bifidobacterium*	↓IL-1β, IL-2, IL-6, TNF-α

IBD, inflammatory bowel disease; TNBS, trinitrobenzene sulfonic acid; DSS, dextran sodium sulfate; DNBS, dinitrobenzene sulfonic; iNOS, inducible nitrogen oxide synthase; COX-2, cyclooxygenase 2; TNF, tumor necrosis factor; IL, interleukin; RORγt, retinoic acid receptor related orphan receptor γt; Foxp3, forkhead box protein 3; TJP, tight junction protein; Th17, T helper 17; T_reg_, regulatory T; ZO-1, zona occludens-1; NF-κB, Nuclear Factor kappa-light-chain-enhancer of activated B cells; TLR, Toll-like receptor; MyD88, myeloid differentiation primary response gene 88; MUC2, mucin2; MPO, myeloperoxidase; p-STAT3, phosphorylated signal transducer and activator of transcription 3; Lgr5, G-protein–coupled receptor 5; CDX2, caudal type homeo box transcription factor 2; NLRP3, NOD-like receptor pyrin domain–containing protein 3; M, macrophage; TGF-β, transforming growth factor-β; LPS, lipopolysaccharides; IFN-γ, interferon-γ; MIP, macrophage inflammatory protein; G-CSF, granulocyte colony-stimulating factor; KC, keratinocyte-derived chemokine; SCFAs, short−chain fatty acids; qPCR, quantitative real-time polymerase chain reaction; 16S rRNA, 16S ribosomal ribonucleic acid.

↓ means reduced; ↑ means increased.

### 3.2 Possible mechanisms of probiotics on regulating gut microbiota-BAs axis in IBD/CAC

In the VSL#3-treated mice, the conjugated/unconjugated BAs ratio decreased, which was relevant to elevated fecal BSH expression and activity compared to the vehicle group. BSH activity was much higher after oral take of *Lactobacillus acidophilus* or *Bifidobacterium infantis* than *Streptococcus thermophilus* (149). Recently, a dual probiotics system including *Lactobacillus delbrueckii* subsp. *bulgaricus* and *LGG* was fabricated, and *LGG* could increase the abundance of BSH-containing gut bacteria (150). Recently, BSH-expressing engineered native *E. coli* could reduce primary conjugated fecal BAs and increase primary deconjugated BAs, consistent with increased BSH activity (151). BioPersist, a bioengineered live biotherapeutic product that enhanced persistence in the colitic mice could reduce the accumulation of BAs, suggesting that BioPersist facilitated efficient BA recycling *via* enterohepatic circulation or improved homeostasis of gut microbial growth (152). As a corollary, it is conceivable that probiotics with BSH can regulate gut microbiota-BAs axis through promoting deconjugation. Simultaneously, they decrease the accumulation of BAs in IBD. Sato and co-authors found that intestinal microbiota participated in 7α-dehydroxylation was restored in distal UC patients after 4 weeks of *Clostridium butyricum* intervention (153). Therefore, it can be inferred that probiotics may modulate gut microbiota-BAs axis by increasing intestinal bacteria involved in deconjugation and 7α-dehydroxylation over the course of BAs biotransformation. The possible elevated gut bacteria at the genus/species level after administration of diverse probiotics were discussed above. Collectively, after using probiotics in IBD and CAC, *Lactobacillus*, *Bifidobacterium*, *Bacteroides*, *Parabacteroides*, *Clostridium*, *Blautia*, *Bacillus coagulans*, *Eubacterium* or *Eubacterium_fissicatena_group* were increased in IBD, and *Blautia*, *Bifidobacterium*, *Lactobacillus*, *Parabacteroides*, *Bacillus*, *Parabacteroides* sp., *Bacteroides acidifaciens*, *Bifidobacterium pseudolongum*, *Clostridium* XI and XVIII were enhanced in CAC. Moreover, the altered BAs profile, especially the likely elevated secondary BAs, were able to bring forth anti-inflammatory and immunomodulatory effects through activating BARs on intestinal immune cells, as reviewed before. In addition to the altered gut microbes after administration of probiotics in these studies, other effects including beneficial immune response were also summarized in the **Tables 1** **–** **3**. Therefore, it could be speculated that the possible altered BA profiles through the administration of probiotics might ameliorate IBD/CAC *via* BAs-BARs–immune cell axis. However, whether probiotics alone and/or their elevated gut bacteria exert beneficial impacts on gut microbiota-BAs axis in the state of IBD or CAC need further explorations of clinical or animal studies. Also, the exact mechanisms are still not elaborated, which require to be appraised in depth. *Lactobacilli* and *Bifidobacteria* are representatives of the main probiotics, spontaneously amass primary, and possibly secondary unconjugated BAs or following intracellular BAs deconjugation in their cytoplasm (154, 155). Sanchez hypothesized that probiotic cytoplasm-sequestered primary BAs will escape being transformed into secondary BAs by other microorganisms. These unconverted primary BAs would be removed with the feces (156). Hence, this BA­accumulation mechanism may make a difference to chronic inflammation (such as IBD) and carcinogenesis (such as CAC).

**Figure 1:**
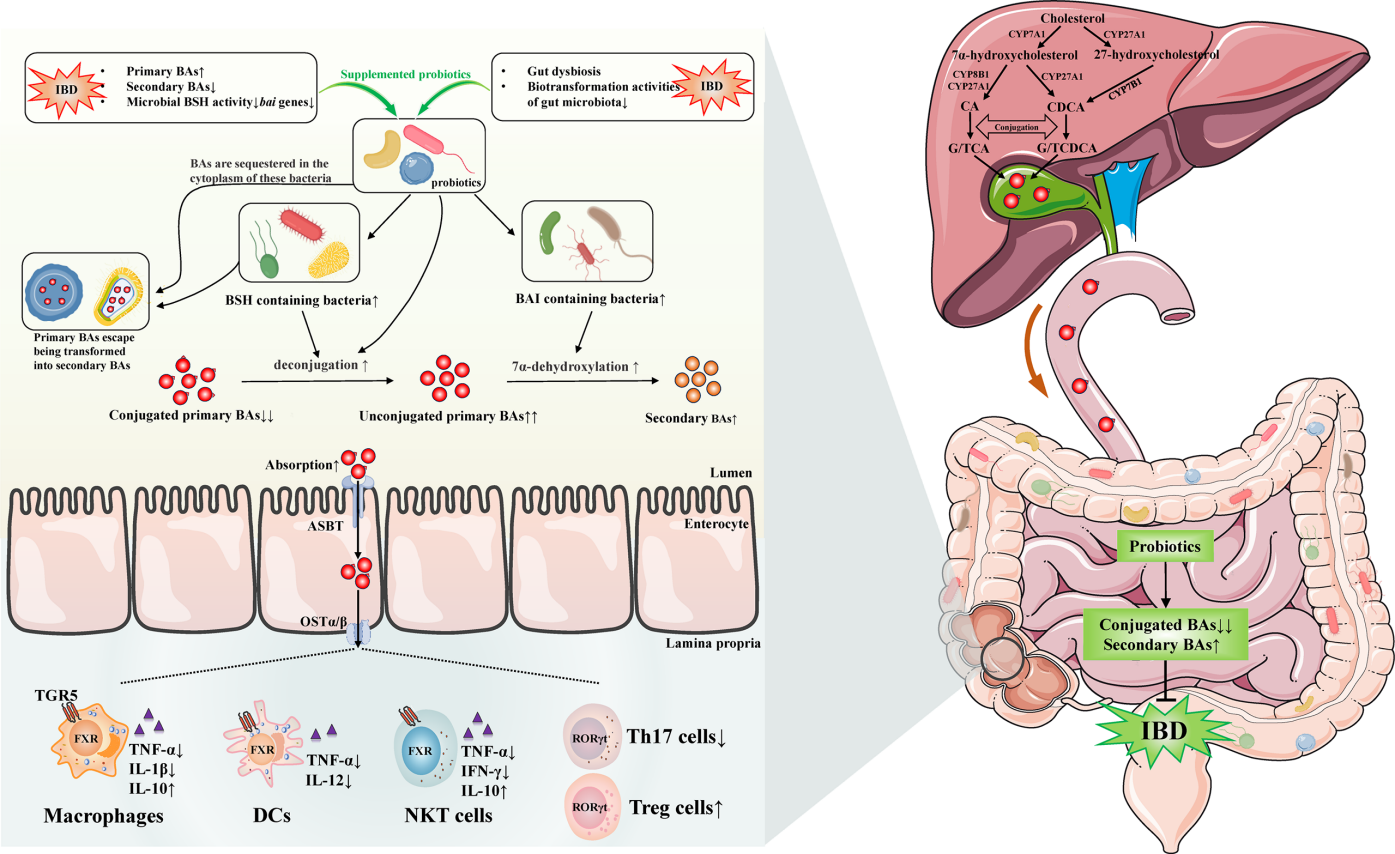
Possible mechanisms of probiotics on regulating gut microbiota-bile acids axis and related gut immunity in inflammatory bowel disease. Gut dysbiosis and BAs dysmetabolism existed in IBD has been discovered by many studies, the levels of secondary BAs are lower, primary BAs are elevated because of the impairment of microbiota biotransformation activities, and the microbial BSH activity and *bai* genes are decreased. The administration of probiotics are likely to increase the BSH and BAI containing bacteria in IBD. Therefore, the conjugated/unconjugated primary BAs ratio decreases and secondary BAs may increase by the elevated activities of deconjugation and 7α-dehydroxylation. Furthermore, probiotics may reduce the accumulation of BAs by facilitating the absorption of them, which are possible to exert beneficial effects by activating BARs on intestinal immune cells, such as monocytes/macrophages cells, DCs, NKT cells, ILC, and T cells. Beyond these, probiotic cytoplasm-sequestered primary BAs may escape being transformed into secondary BAs. These unconverted primary BAs are likely to be removed with the feces. Eventually, the IBD may be improved by these distinct mechanisms after using probiotics. IBD, inflammatory bowel disease; BAs, bile acids; TGR5, Takeda G-protein receptor 5; FXR, farnesoid X receptor; RORγt, retinoic acid receptor related orphan receptor γt; DCs, dendritic cells; NKT, natural killer T; ILC, innate lymphoid cells; Th17, T helper 17; T_reg_, regulatory T; TNF, tumor necrosis factor; IL, interleukin; IFN-γ, interferon-γ; CA, cholic acid; CDCA, chenodeoxycholic acid; GCA, glycocholic acid; TCA, taurocholic acid; GCDCA, glycochenodeoxycholic acid; TCDCA, taurochenodeoxycholic acid; CYP7A1, cholesterol-7α-hydroxylase; CYP8B1, sterol-12α-hydroxylase; CYP27A1, mitochondrial sterol-27-hydroxylase; CYP7B1, oxysterol 7α-hydroxylase; BSH, bile salt hydrolase; BAI, bile acid-inducible enzymes; ASBT, apical sodium-dependent bile acid transporter; OSTα/β, organic solute transporter subunit α/β.

In short, probiotics might involve in gut microbiota-BAs axis by several potential mechanisms, which we mainly focused on the increased gut bacteria containing BSH or BAI at genus/species level in IBD and CAC. We could speculate that probiotics treatment was able to enhance the activity of deconjugation and 7α-dehydroxylation by increasing the BSH- and BAI-containing bacteria in gut of IBD/CAC. Subsequently, the BAs profile was changed, which might include decreased conjugated primary BAs and increased unconjugated primary BAs and secondary BAs. Moreover, probiotics were possible to contribute to the absorption of these altered BAs. Afterwards, the absorbed BAs were likely to generate beneficial effects through activating BARs on intestinal immune cells, such as monocytes/macrophages cells, DCs, NKT cells, ILC and T cells. In addition, probiotic cytoplasm-sequestered primary BAs will escape being transformed into secondary BAs and then be excreted with the feces. Ultimately, the IBD may be improved by these mechanisms after using probiotics (**Figure 1**).

## 4 Conclusions

A large number of studies have revealed the interaction between gut microbiota and BAs. Among them, the intestinal microbiota is involved in BAs synthesis and metabolism, thus influencing BAs composition. Both of which can facilitate the initiation and development of IBD and CAC. Additionally, growing evidence has indicated that certain probiotics exhibit beneficial effects on UC, CAC, and other diseases through multiple mechanisms. In the present review, the possible functional roles of probiotics on gut microbiota-BAs axis were discussed. We laid emphasis on elucidating the alterations of gut bacteria that may involve in gut microbiota-BAs axis in IBD/CAC patients or animal models treated with probiotics. These elevated bacteria at the genus/species level may include *Lactobacillus*, *Bifidobacterium*, *Bacteroides*, *Parabacteroides*, *Clostridium*, *Blautia*, *Bacillus coagulans*, *Eubacterium*, and *Eubacterium_fissicatena_group*. Some mentioned bacteria that may carry some functional genes seem to have the potential to become probiotic for the moment, but whether they can be used as probiotics still needs numerous and rigorous animal researches and clinical trials according to the four criteria for using as probiotics. Moreover, the activation of BARs, including FXR, TGR5, VDR in monocytes/macrophages cells, DCs, NKT cells, ILC, Th17, and T_reg_ cells, exerts anti-inflammatory and immunomodulatory effects, which may partially explain the positive effects of probiotics on IBD. According to American Gastroenterological Association Institute’s advice, probiotics may be considered for the treatment of functional symptoms in IBD. The gut dysbiosis existed in IBD may be improved by using probiotics for a period of time. Due to the biological activity and characteristics of probiotics, there are potential risks in the treatment of IBD patients with probiotics, such as bacteremia, transfer of antibiotic resistance, and adverse reactions. Furthermore, the probiotics used for adjunctive therapy may not be effective in some patients on account of individual difference or strain specificity, so it should be discontinued at this time. Therefore, the duration of probiotics should be determined by different therapeutic goals, effects, and actual conditions. In the future, more clinical and animal studies are necessary to explore the direct changes of BAs and specific mechanisms by administration of probiotics in IBD and CAC.

## Author contributions

LL, YG and HC contributed conception and prepared the manuscript. LL searched the literature and wrote the manuscript. XW, YS and YG prepared the figure. LL, TL, RX and HC revised the manuscript. BW and HC organized the framework. All authors contributed to the article and approved the submitted version.

## Funding

This work was supported by the grants (81970477, 82070545 and 82100574) from the National Natural Science Foundation of China, the Key Project of Science and Technology Pillar Program of Tianjin (20YFZCSY00020).

## Conflict of interest

The authors declare that the research was conducted in the absence of any commercial or financial relationships that could be construed as a potential conflict of interest.

## Publisher’s note

All claims expressed in this article are solely those of the authors and do not necessarily represent those of their affiliated organizations, or those of the publisher, the editors and the reviewers. Any product that may be evaluated in this article, or claim that may be made by its manufacturer, is not guaranteed or endorsed by the publisher.

## Abbreviations

IBD, inflammatory bowel disease; BAs, bile acids; TGR5, Takeda G-protein receptor 5; FXR, farnesoid X receptor; RORγt, retinoic acid receptor related orphan receptor γt; DCs, dendritic cells; NKT, natural killer T; ILC, innate lymphoid cells; Th17, T helper 17; Treg, regulatory T; TNF, tumor necrosis factor; IL, interleukin; IFN-γ, interferon-γ; CA, cholic acid; CDCA, chenodeoxycholic acid; GCA, glycocholic acid; TCA, taurocholic acid; GCDCA, glycochenodeoxycholic acid; TCDCA, taurochenodeoxycholic acid; CYP7A1, cholesterol-7α-hydroxylase; CYP8B1, sterol-12α-hydroxylase; CYP27A1, mitochondrial sterol-27-hydroxylase; CYP7B1, oxysterol 7α-hydroxylase; BSH, bile salt hydrolase; BAI, bile acid-inducible enzymes; ASBT, apical sodium-dependent bile acid transporter; OSTα/β, organic solute transporter subunit α/β.
